# Is a General Register for Nurses Desirable?

**Published:** 1888-07-28

**Authors:** Henry Bonham-Carter

**Affiliations:** Honorary Secretary, the Nightingale Fund.


### Is a General Register for Nurses Desirable?

By Mr. Henry Bonham-Carter, Honorary Secretary, The Nightingale Fund.

Judging by the public and private discussions which have taken place among members of the nursing profession and the expression of opinion of some medical men, it would seem to be a foregone conclusion on their part that this question can only be answered in the affirmative, the matter for discussion and any differences of opinion having apparently had relation only to the method and machinery to be adopted in establishing a Register.

I propose to submit for consideration some reasons on the negative side of the question tending to the conclusion that the object which all who are interested in the promotion of good nursing have at heart, namely, that of raising the standard of nursing and the position of nurses, may suffer instead of gaining by the establishment of a general Register. The advantages to be obtained by it have been stated from the nurses' point of view in words to this effect, "that nursing is a profession as medicine and law are professions; but that it is not acknowledged as such; and that the legal registration of nurses is the only means by which nursing can be established as an acknowledged and legally-constituted profession; that to effect this a Royal charter must be obtained, which will authorise the registration of nurses on terms satisfactory to physicians and surgeons as evidence of their having received systematic traning.

From the doctors' point of view it is stated that the medical profession require some definite means of knowing with certainty who is and who is not a trained nurse; that in the medical and other professions a legal Register provides that means; and that it is hardly necessary to point out that unless such a Register be authorised by the State it is of no value; that it is essential, moreover, that the Register should be controlled by the medical profession.

Both, therefore, the nurses and the doctors whose opinions have been quoted concur in the view that the case of nurses is analogous to that of the medical profession, and while the nurses consider that a legal Register is essential, in order to raise the position and vocation of a nurse to that of a profession, with all the supposed advantages attaching to that designation, the doctors are of opinion that it affords the only safe means by which to provide themselves with a skilled nurse. Now, I venture to think that, when we come to look more closely into the matter, we shall find some patent reasons for concluding that the case of nurses is not analogous to that of doctors, that a general Register will not afford and is not capable of affording, either to doctors or to the public, the information which employers of nurses ought to require, and, lastly, that it may tend rather to lower than to raise the position of nurses.

In the case of the medical profession, the Register is merely a certificate of the candidate having passed certain examinations, after a certain period of technical training, and it is supposed to be required, partly on the ground that the public require protection by precluding unskilled persons, quacks, and others, under penalties, from practising medicine at all, and partly on the ground that, medical men having to perform certain public duties imposed upon them by law, a legal certificate of their qualification is expedient.

The qualification for the Register is based mainly, if not solely, on the result of examination, and affords a guarantee only of a certain amount of professional knowledge and skill, coupled with some evidence of previous good conduct, or, rather, absence of bad conduct. The machinery of a general Register is not, and does not pretend to be, adapted for attaining any result of a more thorough or discriminating kind as far as character is concerned. No one can be removed from the Register except for gross negligence or misconduct, and the removal has to be effected by means partaking of the character of a legal process.

How far a legal Register is essential or advantageous in the case of doctors I will not stop to inquire. Some of the highest authorities in the profession have expressed more than doubts on the subject. But let us consider what are the qualifications which are required of a good nurse, and how far these can be satisfactorily ascertained after the manner in which a medical student's qualifications are tested. No one will, at this time of day, deny that moral as well as professional qualities are everything in a nurse, that she has to be judged by her character and conduct as well as by her technical skill, by the possession of such qualities as kindness, patience, trustworthiness, self-control, discretion. How are these intangible things to be registered?

The whole history of the movement for the improvement of nursing affords a protest against the assumption that the case of nurses is, in this respect, analogous to that of medical students. The main efforts of those who have promoted or carried out the better organisation of hospital nursing, and have established and superintended training schools for nurses, have been directed to the introduction of such a system as would protect and foster the moral qualities of the nurse as being an essential accompaniment to the provision of the requisite technical instruction. It was felt that the efficiency of the nurse was to be gauged first by the possession of these qualities, without which the professional knowledge and acquirements were comparatively useless for all practical purposes. The conduct and the behaviour, as well as the practical skill of the nurse, were, as shown in her daily work in the wards, to be continuously observed and reported on, so that means should be afforded of effectively correcting deficiencies, and testing progress in both. The final estimate of the nurse's qualifications was held to depend in a large measure on the possession in greater or less degree of these moral qualities, which are not ascertainable by examinations nor measurable by marks.

The nature of the nurses' duties, and the training and qualifications required for fulfilling those duties, render the case of nurses in this respect very different from that of medical students.

To refer now to the machinery of the Register.

It is not enough to say that the moral character of the nurse can be ascertained by a certificate of good conduct from the training school previous to admission upon the Register; for in the first place who is to certify the training school? and, in the next place, how is the evidence of the character to be kept up in subsequent years after the name has been placed on the Register?

Training schools vary greatly in their character. The institution may be said to be, as regards by far the greater number of them, of comparatively recent date. Their methods are different, their standards of requirements are unequal, and although very considerable progress has been made, yet very much still requires to be done. It would be useless to attempt to force the growth of these institutions towards more advanced views by any legislative measures. Moreover, it cannot, I venture to think, yet be said that those who have the direction of hospitals and training schools, whether laymen or professional men, have arrived at such a general concurrence of opinion as to the system to be pursued in the school and hospital, or in the standard of qualification to be required of a nurse, as will render it practicable or expedient to lay down any general conditions to which training schools should conform in order that their pupils may be entitled to admission on a general Register.

Then comes the further difficulty as to keeping up the value of registration.

Nurses are not exceptional in being subject to great deterioration if not kept up to the mark by pressure from outside, and the character of their work renders them more subject to temptations and vicissitudes tending to deterioration than others.

In every-day life, those who know what they are about, require, before engaging a nurse, recent evidence, and evidence which must be of a confidential kind, as to moral qualities and the nature and degree of these qualities. Is it possible that the machinery of a legal Register can be made capable of obtaining and affording such information? The nature of the evidence required and the legal character of the Register appears to preclude any such result.

But it is said that the public does not take its doctors from the Register, nor will it take its nurses; that the Register is only required as a safeguard, a preventitive against untrained nurses being employed by a necessarily ignorant public, that the public and medical men will thus gain great benefits, and may still continue to require further evidence of a private character.

To this it may be safely answered that there cannot be a doubt that the great mass of the public, as well as of medical men, will be content to take their nurses from the Register and the Register only; and even assuming that it may be of value at first the effect will be that in the course of no long time after its establishment the Register will become a very untrustworthy guide.

Now I come to this further consideration. It is the opinion of many of those who have had the longest and widest experience as matrons of hospitals and heads of training schools, that the effect of a general Register upon the standard of training will have the very reverse effect to that contemplated by its supporters, namely, to lower instead of raising the standard. This result must be a necessary consequence if my previous contention be correct that the real character of the nurse will not find a place on the Register, for if the Register is merely based upon the possession of technical knowledge, accompanied by a colourless certificate of good conduct, the value of those moral qualities to which so much importance attaches as a test of efficiency, will be entirely lost.

In the next place, it is obvious that the conditions for admission on the Register must be so framed as to conform to the capabilities of the average nurse and to meet the requirements of the average training school. Great injustice would be done to a number of respectable women were a higher standard insisted on, and seeing that the official recognition of the Register would be considered sufficient by the great mass of employers, the result would be that the same advantages would be thereby conferred on the inferior as on the superior nurse. It would also thus tend to destroy any ambition on the part of the bulk of nurses to obtain the higher certificate which might be afforded by a superior over an inferior training school, and lead to a general and low uniformity of qualification.

I am permitted to quote upon this subject the opinion of an experienced hospital matron, who writes as follows, in reply to an inquiry as to her views upon the establishment of a general Register with certificate based upon examination: "I think both these things, viz., the examination of, and the granting certificates to, nurses, are most necessary, but that both should be done at the school where the nurses have been trained, or in many cases gross injustice will be done to those best fitted to become nurses, and immense harm to the profession itself. At the present time nursing is fashionable, and there cannot be the slightest doubt that there are large numbers of young women taking it up, not because they have a taste for it, not because they wish to be useful in this life, not even because they desire to earn their livelihood, but simply because they have an idea that when once their probation is over they will lead a life of much greater freedom than they possibly could if they continued to reside with their friends, and in this they are right; now these are the women who will most easily pick up the scientific part of the work, and who will, in consequence of their cleverness, their better education, their power of putting their knowledge into words, most certainly come out well in all examinations, leaving far behind the less well-educated and clever ones, although the knowledge of nursing possessed by these may be quite as great, and their aptitude and fitness for their work much greater. Therefore, the clever and unfit ones will obtain first-class certificates, and, consequently, in many instances, good posts, while the others will only obtain second-class certificates and inferior posts, which will be an injustice to them and a great loss to institutions and the nursing profession generally. The granting of the certificates by the authorities where the nurses have been trained is the only means I know of for preventing this evil, because they alone can know what sort of nurses and what sort of women the candidates for examination are. It will be useless to say that the candidates must be recommended by their matron or superintendent, for the women I describe never give the authorities any particular cause for complaint during their probation, therefore, they would have to be recommended, but anyone can make a fairly-accurate guess as to how far they will do credit to their training when once they are free from control."

Then there are important considerations arising out of the legal character of the proposed Register. One phase of these arises out of the liabilities which hospital authorities might incur by dismissing a registered nurse or even a probationer nurse with or without cause assigned. Another is the difficulty of removal from the Register. The first was referred to by a writer in the *Times* at an early stage of this discussion.

A probationer nurse is discharged on the ground of want of aptitude for the work, inefficiency, or misconduct. She is thereby in all probability deprived of the power of qualifying herself for admission on the Register or becoming a member of a profession endowed with certain privileges. This is a much more serious loss than would be incurred by the deprivation of the power of gaining a private certificate from any given hospital, and might entail actions for wrongful dismissals or libel. Then, with regard to the discharge of a Registered nurse not only do the same difficulties arise, but besides this, the mere fact of being on the Register would tend to make her independent of the certificate of character to be obtained from the hospital itself which employs her, and would be likely to give rise to serious difficulties in the disciplinary control of the staff.

The second objection is that of the impossibility of removing a nurse from the Register where the grounds for removal have arisen from moral defects which do not amount to gross and patent misconduct, and yet are such as to render the nurse unfit for her vocation. Any removal from a public Register must rest on evidence of a public and not of a confidential and privileged character, and it is obvious that no person would come forward to give open evidence with reference to defects of the kind referred to. It would be nobody's interest to do so, having regard to the liabilities which would be thereby incurred.

These objections apply equally to the proposal to require a periodical renewal of some sort of certificate of character and conduct.

They do not, however, apply to Registers kept by hospitals or training schools; nor necessarily, or at least in the same degree, to Registers established by voluntary associations not having any legal character.

In considering this question it must be borne in mind that the class of persons to be dealt with will never be composed mainly, or even largely, of matrons, superintendents, ward sisters, and others, holding superior appointments. To these, indeed, the Register would be of no particular advantage so far as advancement in their vocation is concerned, nor even would it to the rank and file who are engaged in hospital service, so long as they so continue. It is with those who are engaged in private nursing that the Register would have mainly to deal, and these are at present, and in this country probably always will be principally, if not entirely, recruited from the lower middle class, who, from education and social position, cannot be greatly under the influence of public opinion.

This consideration affords a further and potent reason against the existence of the supposed analogy between the nursing and the medical profession.

As to the allegation that a legal Register or so-called legal status is essential in order to afford to nu ses the advantages of an acknowledged "profession," it may be observed that the position which nurses have already gained has been due to their own efforts in taking advantage of the opportunities afforded to them, and to the just appreciation with which the public and the medical profession have regarded the results attained. Have these causes come to a standstill? Is there any reasonable ground for supposing that any legislative sanction would raise or improve their position? There is yet very much to be done before the acknowledged defects and inequalities which are still to be found in the training and education of nurses shall have been removed; but progress and improvement are to be looked for from the same sources as heretofore, voluntary effort and public opinion.

It is not necessaiy for my argument to touch upon the claim, to which reference is made at the beginning of this paper, as having been put forth from the doctors' point of view, that the Register should be controlled by the medical profession. I will only remark that such a contention would seem to be based on the same erroneous view of the subject as that which treats the case of nurses as analogous to that of medical students.

In conclusion, I would add that even should the foregoing remarks not have convinced those who are in favour of the establishment of a public Register, yet they may, at any rate, have the effect of leading to the conclusion that the time is not yet ripe for any such measure.

